# Bilateral synchronous UBE for unilateral laminotomy and bilateral decompression as a potentially effective minimally Invasive approach for two-level lumbar spinal stenosis

**DOI:** 10.1038/s41598-025-86106-8

**Published:** 2025-01-20

**Authors:** Yulin Zhao, Yingjun Guo, Xin Pan, Hao Li, Xianlei Gao, Haipeng Si, Wanlong Xu

**Affiliations:** 1 Department of Orthopedics, Qilu Hospital of Shandong University (Qingdao), Cheeloo College of Medicine, Shandong University, Qingdao, 266035 Shandong, PR China; 2 Department of Orthopedics, Qilu Hospital of Shandong University, Cheeloo College of Medicine, Shandong University, Jinan, 250012 Shandong, PR China; 3 Key Laboratory of Qingdao in Medicine and Engineering, Department of Orthopedics, Qilu Hospital (Qingdao), Shandong University, Qingdao, 266035 Shandong China

**Keywords:** UBE, ULBD, Lumbar spinal stenosis, Endoscopy, Neuroscience, Anatomy, Medical research

## Abstract

Currently, Unilateral biportal endoscopy is widely used in the surgical treatment of lumbar spinal stenosis. To investigate the feasibility of bilateral synchronous UBE to unilateral laminotomy and bilateral decompression(BS-UBE-ULBD) for treating two-level lumbar spinal stenosis (LSS). Sixty-four patients with two-level lumbar spinal stenosis (LSS) treated with BS-UBE-ULBD from October 2022 to January 2024 were retrospectively analyzed. All patients were treated with BS-UBE-ULBD. All 64 patients successfully underwent surgery, and the duration of surgery was 95–180 min, with an average of 119.92 ± 14.79 min. The average number of fluoroscopy was 3.02 ± 0.92. The average blood loss during the surgery was 73. 44 ± 36.70 ml. Postoperative lumbar CT showed that the spinal canal and bilateral nerve roots were fully decompressed. There were no postoperative complications, such as infection, severe nerve root injury, and lumbar instability. Complete follow-up data were obtained for all 64 cases. The VAS score of low back and leg pain and the ODI of lumbar function significantly (*P* < 0.05) improved at each follow-up time point. MacNab evaluation at 6 months after the surgery showed that the results were excellent in 48 cases, good in 14 cases, and fair in 2 cases. The excellent and good rate was 96. 88% (62/64). So BS-UBE-ULBD is a minimally invasive, highly effective, and safe procedure for 2-level LSS.

Degenerative lumbar spinal stenosis (LSS) refers to clinical symptoms caused by the compression of the cauda equina, nerve root, and vascular complex. LSS can occur due to the abnormal shape and volume of the bony or fibrous structure after degenerative changes and the stenosis of the inner diameter of one or more lumens at a single level or multiple levels. It is a common cause of lumbago or lumbago and leg pain, which is common among middle-aged and elderly people^1^. LSS has become the most common cause of lumbar surgery among patients over 60 years of age^2^. However, traditional surgery necessitates extensive stripping of paraspinal muscles, which can easily lead to the ischemic injury of paraspinal muscles and atrophy after denervation. Therefore, traditional surgery may result in intractable back pain, stiffness, and discomfort after surgery^3,4^. Furthermore, as the posterior bone and soft tissue structures need to be extensively resected during the surgery, epidural scar and nerve compression are highly likely after the surgery. The high risk of general anesthesia cannot be ignored among elderly and weak patients^2,5^.

Recently, with the rapid development of minimally invasive spine surgery, endoscopic surgery has been applied in the treatment of LSS^6^. Unilateral biportal endoscopy (UBE ) is more popular in treating LSS and is a more flexible operation, with small trauma, quick recovery, and a gentle learning curve. In addition, many studies have proven the good clinical efficacy of unilateral biportal endoscopy^7^. Multilevel spinal stenosis can be done simultaneously. Previously, the same operator decompressed multiple segments in turn^8,9^, but the operation lasted longer, and the corresponding problems, such as bleeding, high risk of anesthesia, and fluoroscopy frequency, increased.

From October 2022 to June 2024, our hospital pioneered the use of Bilateral Synchronous UBE-unilateral Laminotomy and Bilateral Decompression (BS-UBE-ULBD) for two-level degenerative LSS. Sixty-four patients with two-segment LSS were treated with BS-UBE-ULBD, and the results were satisfactory.

## Data and method

### General information

A retrospective analysis was performed on 65 patients with bilevel LSS admitted to our hospital (Qilu Hospital of Shandong University, Jinan) between October 2022 and January 2024.They were treated with UBE-ULBD under double-channel endoscopy on both sides. There were 34 males and 31 females, aged 57–82 years, with an average age of (66.88 ± 6.42) years, and the disease course lasted 6–120 months (mean ± SD: 36.13 ± 25.77 months). One patient died of respiratory failure due to the novel coronavirus pneumonia infection 5 months after the operation and was lost to follow-up. All patients were given symptomatic treatment before the operation until there was no obvious surgical contraindication. The inclusion criteria were as follows: ① the patients failed to respond to conservative treatment for more than 3 months; ② the symptoms and signs were consistent with the results of imaging examination, and all of them were confirmed as double-segment LSS; ③ patients and their family members understood the complications of the operation and signed the informed consent form; ④ the patients completed the follow-up process after the operation. The exclusion criteria were as follows: ① patients with lumbar lesions affecting 3 or more segments; ② patients with severe stenosis of intervertebral foramen; ③ patients with lumbar instability or lumbar spondylolisthesis above II °; ④ patients with a history of lumbar surgery or infection, tumor, and severe systemic medical diseases.

### Method

**Fig. 1:**
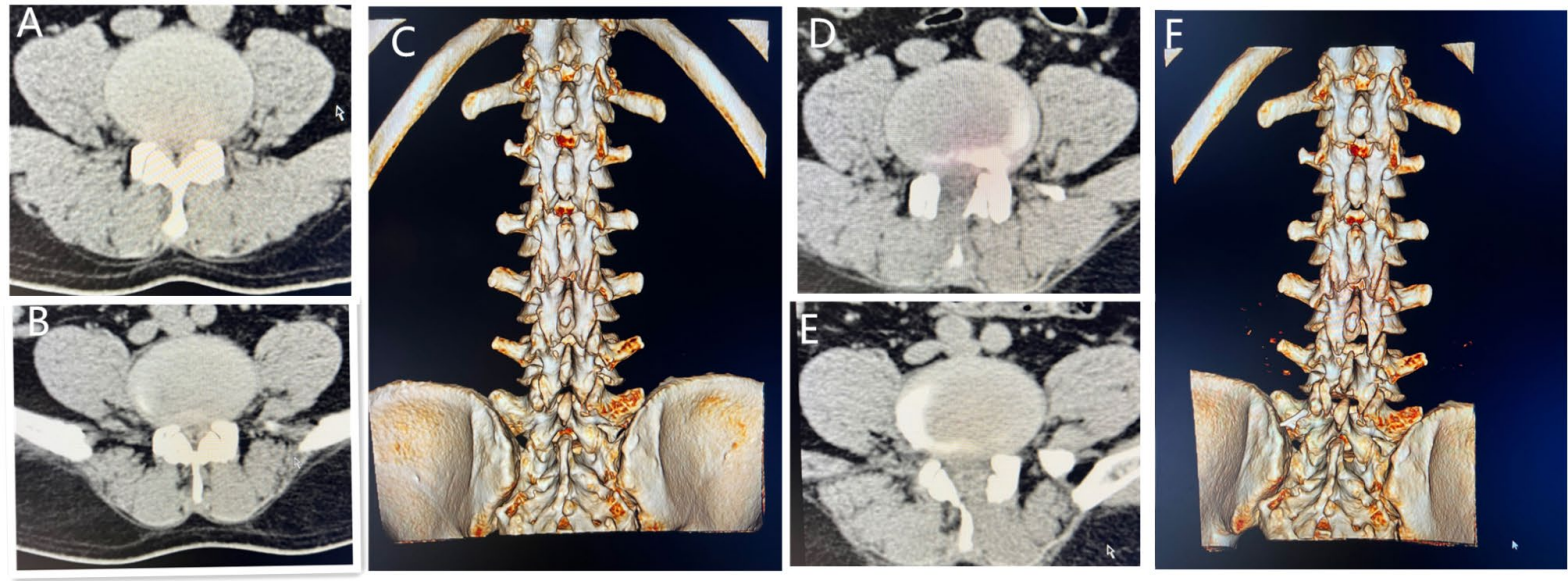
A: Determining the position of L3/4/5 pedicle of vertebral arch and target point by body surface fluoroscopy; B: Determining the position of intervertebral space by simultaneous fluoroscopy of two segments; C: standing position of the operator; D: the intraoperative anteroposterior fluoroscopic positioning view.

After successful general anesthesia, patients were placed in the prone position, and two sets of spinal UBE instruments were routinely prepared. The patient was placed in the prone position, and the bed surface was adjusted to make the target intervertebral space as vertical as possible relative to the ground. The lesion site and the corresponding intervertebral space level were determined by fluoroscopy (Fig. 1, AB). Two operators treated one segment on each side of the patient, The two surgeons are from the same center, the same diagnosis and treatment team, and are doctors of the same level and level. and routinely incised with a sharp knife at the inner edge of the pedicle 1 cm from the spinous process and 1.5–2 cm above and below the intervertebral space. The endoscope and working channel were determined based on the surgeon’s left and right-hand operation habits (Fig. 1, AB). Operator’s station (Fig. 1, C); a serial dilator was used for insertion into the lamina. The working cannula was placed after removing the dilator. The vertebral plate was opened in the working channel for operation. Figure 1,D shows the intraoperative anteroposterior fluoroscopic positioning view. Based on the location of the lesion, ipsilateral hemilaminectomy was conducted using a drill and a rongeur to expose the deep part of the ligamentum flavum. The drill and the rongeur were used to remove hypertrophic facet joints and lamina. Then, the ligamentum flavum and the dural space were explored using a blunt hook to ensure that there was no adhesion. The ligament and nerve were stripped using a curette and a rongeur to decompress. For contralateral decompression, the midline of the spinal canal was first determined using a high-speed drill. The range was then adjusted from the middle. Partial resection of the base of the spinous process prevented it from obstructing the operating range to ensure adequate working space. After exposure, the ligamentum was dissected from the contralateral lamina and cut. The contralateral approach was performed dorsally to the dura, keeping the ligamentum flavum intact. The craniocaudal laminotomy was used for additional decompression. Partial resection of the contralateral superior articular process was conducted to preserve the integrity of the facet joint. After complete decompression of the bony structure, the hypertrophic ligamentum flavum was resected to fully decompress the nerve structure. The endpoint of decompression was the outer edge of the bilateral nerve roots^10^. Bilateral consistent operation was conducted. Drains were placed bilaterally.

Dehydration, detumescence, nerve nutrition, and other drugs were used after the operation. On the first day, the patients began to get out of bed under the protection of hard waistline fixation. The drainage was removed within 24 h after the surgery. Within one month after the operation, the patients mainly rested in bed and properly exercised the back muscles. Patients are encouraged to get up and move around early but are not encouraged to participate in physical exercise. Three months after the surgery, the patients were forbidden to do heavy physical activity involving the waist. Postoperative guidance was provided to strengthen the training of back muscle strength, correct inappropriate living habits, and reduce the recurrence rate.

This study was conducted in accordance with the principles of the Declaration of Helsinki. The ethics committee of our center approved this study. We obtained informed consent from all participants in this study. This study report conforms to the PROCESS standard^11^.

### Observation index

The operation time, fluoroscopy times, intraoperative blood loss, postoperative hospital stay, and lumbar Oswestry disability index (ODI) were recorded before the surgery and 1 week and 6 months after the surgery. Modified MacNab was used at 6 months postoperatively^12,13^.

### Statistical analysis

Statistical Analysis IBM SPSS Statistics ver. 22.0 (IBM Co., Armonk, NY, USA) was used for statistical analysis. A paired t-test was used to compare the VAS and ODI scores of low back pain and leg pain before surgery and 1 week and 6 months after surgery. The mean values ​​were expressed as standard deviations, and the Student-t test was used to analyze the differences between the two groups. A p-value of < 0.05 was considered statistically significant. Intraclass correlation coefficient (ICC) was used to evaluate the intrarepeatability of different observers (interobserver reliability). One independent researcher blinded to the group allocation completed the evaluations.

## Results

Results 1: There were 34 males and 31 females, aged 57 to 82 years old, with an average of 66. 88 ± 6.42 years old. The disease course was 6 to 120 months, with an average of 36.13 ± 25.77 months. In total, 64 patients were followed until 1 week and 6 months after the surgery. There were 1 case of L2/3 and L3/4 involvement, 17 cases of L3/4 and L4/5 involvement, 1 case of L3/4 and L5/S1 involvement, and 45 cases of L4/5 and L5/S1 involvement. All patients had neurogenic intermittent claudication with or without radicular pain in the waist and lower extremities. Twenty-two patients were complicated with hypertension, 9 with diabetes mellitus, and 7 with coronary heart disease.

Results 2: The surgery was successfully completed for 64 patients. The surgical time was 95 ~ 180 min, with an average time of 119.92 ± 14.79 min. The number of fluoroscopy was 2 to 5 (average 3.02 ± 0.9). Intraoperative blood loss ranged from 50 mL to 150 mL, with an average of 73.44 ± 36.70 ml. The mean postoperative hospital stay was 4. 06 ± 0.96 days (2 to 7 days) (Basement characteristics, Table 1). No complications, such as infection, poor wound healing, and epidural hematoma, occurred during follow-up. One patient had pain and discomfort in the contralateral lower limb after lamina fenestration and was treated with neurotrophic, anti-inflammatory, and analgesic conservative treatment after the surgery. The patient’s symptoms were significantly improved 2 months after the surgery. One patient had a dural tear of nearly 2 mm during the operation. Cauda equina herniation did not occur, which was repaired under the microscope. The skin incision was tightly sutured, and the patient was kept in bed for one week after the surgery. No special discomfort was found, and the incision healed normally. No antibiotics were prescribed postoperatively.

**Table 1:** Basement characteristics.

Items		Data (*n* = 64)
Sex (M/F)		34/31
Age (years)		66.88 ± 6.42
Disease duration (months)		36.13 ± 25.77
Levels	L2-L4	1
	L3-L5	17
	L4-S1	45
	L3/4 + L5/S1	1
Operation duration(M)		119.92 ± 14.79
Number of X-ray shots		3.02 ± 0.92
Blood loss volume(ml)		73.44 ± 36.70
Length of stay(d)		4.06 ± 0.96

**Fig. 2:**
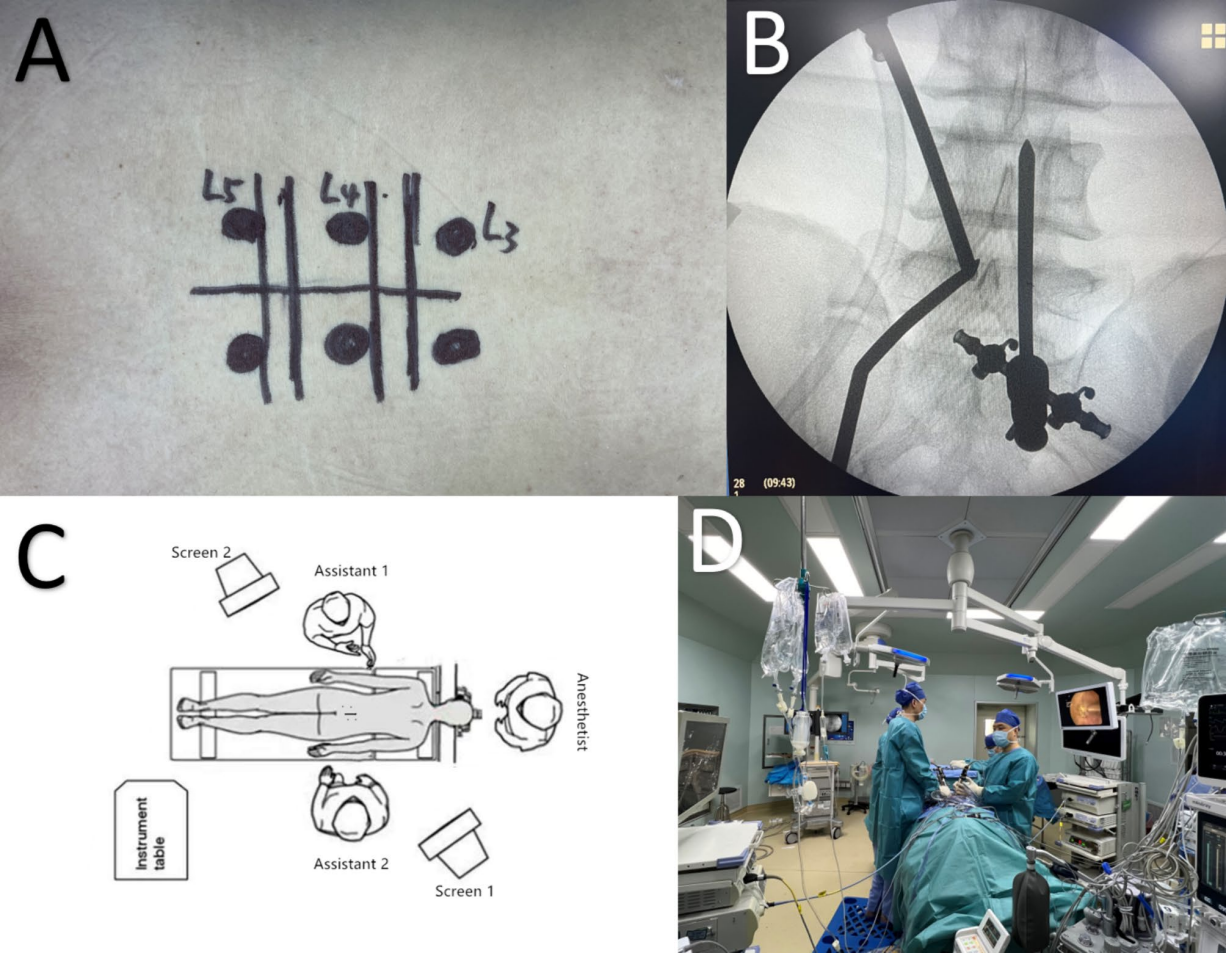
Case 1; Male, 54 years old, lumbar spinal stenosis L3 ~ 5, L3/4 right approach decompression, L4/5 left approach decompression, bilateral simultaneous. A: L3/4 preoperative CT, B: L4/5 preoperative CT, C: preoperative CT reconstruction; D: L3/4 postoperative CT, E: L4/5 postoperative CT, F postoperative CT reconstruction showed that the right L3/4 and the left L4/5 laminae were missing.

Results 3: None of the 64 patients experienced the recurrence of their symptoms during the follow-up period. The VAS scores and ODI of the waist and leg at 1 week and 6 months after the surgery were better than those before the surgery (Table 2). Lumbar VAS (*P* < 0.05), leg VAS (*P* < 0.05), and ODI (*P* < 0.05) at 6 months were better than those at 1 week. At 6 months follow-up, 56 patients reported disappearance of intermittent claudication, and 8 patients still had claudication, but their claudication significantly improved compared with their preoperative claudication. Based on the modified MacNab criteria, the results were excellent in 48 cases, good in 14 cases, and fair in 2 cases, and the excellent and good rate was 96. 88% (62/64)( Typical Cases, Fig. 2).

**Table 2:** Comparison of the VAS score and ODI of waist and leg in 64 patients before and after the surgery (X ± s).

Indicators	Preoperative	One week after surgery	P1	6 months after surgery	P2
Lumbar VAS score/point	4.59 ± 0.77	1.97 ± 0.64	< 0.05	1.22 ± 0.55	< 0.05
Leg VAS score/point	5.42 ± 0.89	1.95 ± 0.89*	< 0.05	1.16 ± 0.76	< 0.05
ODI	34.19 ± 3.48	17.41 ± 2.65	< 0.05	12.17 ± 1.58	< 0.05
VAS: visual analogue scale; ODI: lumbar Oswestry disability index; *P1*, the P-value between 1-week postoperative and preoperative; *P2*, P-value between 6-month postoperative and preoperative					

## Discussion

Surgical treatment aims to relieve nerve compression in the spinal canal^14,15^. Conservative treatment is considered the first line of treatment and includes oral analgesics, anti-inflammatory agents, behavioral improvement, and physical therapy^16^. Additional injectable medications may also be administered to selectively achieve nerve root block or epidural block, although their effectiveness remains inconsistent^17,18^. Although traditional open surgery can relieve spinal canal compression, the surgical trauma is large, and there may be postoperative discomforts, such as waist and leg pain and discomfort, muscle stiffness, and iatrogenic lumbar instability^19,20^. These discomforts delay the postoperative rehabilitation of elderly patients and increase their mental burden; thus, many patients give up treatment, and their quality-of-life decreases. Related studies^21–24^have reported that for LSS without significant lumbar instability, lumbar spinal canal decompression alone can achieve satisfactory results without the need for more invasive fusion. With the rapid development of spinal endoscopy, UBE has been gradually applied to the treatment of various spinal diseases. In this study, lumbar instability and spondylolisthesis of degree II and above were excluded, and ULBD was conducted for 60 patients under dual-channel endoscopy. It belongs to simple spinal canal decompression and conforms to the current concept of minimally invasive surgery. It can minimize intraoperative trauma and blood loss and shorten postoperative recovery without affecting the quality or degree of bone decompression^10,25^ .

As early as 2002, Khoo et al.^26^. reported the application of MIS-ULBD in the treatment of lumbar spinal stenosis. Spinal surgeons attempt to apply MIS-ULBD to the field of endoscopy. The application of spinal endoscopy in the treatment of LSS with dual-channel UBE-ULBD has become a relatively mature technology. With the help of endoscopic laminectomy rongeur and endoscopic high-speed drill, physicians use total spine endoscopy to effectively and rapidly decompress the central spinal canal, bilateral lateral recesses, and bilateral intervertebral discs using the “overhead decompression” technique. This strategy can better release the dural sac and bilateral nerve roots^27^. The UBE-ULBD technique minimizes the difficulty of surgery^18,28,29^. It has attracted the interest of many spine surgeons and has been reported for multilevel spinal stenosis^8,9^. However, currently, most physicians still choose two-segment decompression in turn for two-segment stenosis. To shorten the duration of surgery and improve safety, two physicians bilaterally and simultaneously decompressed the two affected segments in this study.

In this study, the excellent and good rate of 64 cases of two-segment LSS was 96.88% after 6 months of follow-up. There were no complications, such as infection, poor wound healing, and epidural hematoma. The VAS scores and ODI of the waist and leg at 1 week and 6 months after the surgery were significantly better than those before the surgery (*P*< 0.05). Compared with the sequential multi-segment decompression in previous studies^9^, simultaneous bilateral decompression significantly shortened the operation time, reduced the operation and exposure time, reduced intraoperative bleeding and radiation exposure, and reduced the length of hospital stay. Two sets of UBE instruments need to be prepared in BS-UBE-ULBD, and the body position and instrument placement need to be planned in advance to avoid interference between the two groups of operators. This study shows that although the cost of anesthesia and intraoperative fluoroscopy has decreased, the overall surgical cost is still higher than the traditional two-segment UBE-ULBD surgery due to the increased demand for surgical consumables and equipment. However, a mere increase in cost does not indicate a lack of economic benefit from the new technology. It is worth noting that although the cost of the operation is slightly higher, the significant shortening of operation time and anesthesia time and the reduction of patient radiation risk bring obvious benefits to patients. ① New technology can effectively reduce the time of surgery and anesthesia, improve patient comfort and satisfaction, which may reduce the occurrence of postoperative complications and promote faster recovery. ②Reduce the radiation exposure suffered by patients during surgery, which reflects the hospital’s sense of responsibility for patient health. ③Although the total cost of surgery has increased, when combined with multiple factors such as operative time, anesthesia time, risk reduction, and patient safety, this modest cost increase can be regarded as a cost-effective investment. Taking the above factors into consideration, the modest increase in surgical costs is worth the resulting benefits, especially in the context of improved long-term patient outcomes and safety, and this investment is completely justified.

Currently, there are some shortcomings, and we will explore and discuss the following shortcomings in future studies: ① the follow-up time of this study was short, and only two follow-up time points were set, thus long-term efficacy still necessitates further studies; ② there no control group in this study, and objective data were lacking.

To sum up, BS-UBE-ULBD can effectively treat all clinical symptoms of patients with two-level degenerative lumbar spinal stenosis on the premise of ensuring clinical safety. The BS-UBE-ULBD technology has a good development prospect in the treatment of two-segment or even more segmental degenerative lumbar spinal stenosis.

## Data Availability

The data that support the findings of this study are available from the corresponding author, Si ，Xu upon reasonable request.
